# The accuracy of ultrasonography in the diagnosis of superficial bladder tumors in patients presenting with hematuria

**DOI:** 10.4103/0256-4947.51802

**Published:** 2009

**Authors:** Konstantinos Stamatiou, Ioannis Papadoliopoulos, Stefanos Dahanis, Grigoris Zafiropoulos, Konstantinos Polizois

**Affiliations:** a Department of Urology, General Hospital of Thebes, Thebes, Greece; b Department of Radiology, General Hospital of Thebes, Thebes, Greece; c Department of Urology, Thriassio General Hospital, Elefsina, Greece

## Abstract

Ultrasonography has been proposed as the initial test for detection of bladder carcinomas in patients presenting with hematuria, but the accuracy of transabdominal ultrasonography in the diagnosis of superficial bladder carcinoma has not been assessed. We prospectively evaluated 173 patients presenting to the outpatient department with painless hematuria by transabdominal ultrasound and cystoscopy. The tolerability of cystoscopy was also assessed. Of 148 patients who met the inclusion criteria, 39 with bladder carcinoma were identified by cystoscopy as having bladder carcinoma, while 34 were identified by ultrasonography. For ultrasonography, the sensitivity (87.1%), specificity (98.1%), positive predictive value (94.4%) and negative predictive value (95.4%) were good but not as good as cystoscopy. While the tolerability of cystoscopy is relatively low, it is still superior to ultrasonography in the evaluation of the bladder as a possible source of hematuria.

Painless hematuria usually is the sole presenting symptom in the majority of patients with bladder cancer.^1^ Therefore, painless hematuria of any degree in adults should be regarded as a symptom that is suspicious for malignancy until proved otherwise. Cystoscopy is considered the gold standard in the evaluation of hematuria.^2^ This technique directly visualizes lower urinary tract anatomy and macroscopic pathology, which may be responsible for the clinical picture under evaluation. However, cystoscopy is invasive, time-consuming and expensive.^3^

Currently, modern sensitive transducers have improved imaging of the urinary tract and therefore transabdominal ultrasound is more effective in visualizing intra-luminal filling defects in the bladder than it was in the past. Moreover, ultrasonography is a non-invasive, well-accepted, and cost-effective diagnostic procedure. Systematic use of ultrasonography has been proposed as the initial test for detection of bladder carcinomas in patients presenting with hematuria.^4^ The present study was carried out to investigate prospectively the accuracy of modern transabdominal ultrasonography in the diagnosis of superficial bladder carcinoma. The tolerability of cystoscopy was also measured. To our knowledge, no trials have compared the accuracy of modern ultrasound devices with cystoscopy in the diagnosis of superficial bladder carcinoma and only a few studies have given attention to the tolerability and acceptance of urological endoscopic procedures by patients.^5^–^8^

## PATIENTS AND METHODS

In this controlled prospective study, which took place at the General Hospital of Thebes (Viotia, Greece) from April 2006 to January 2008, 173 consecutive patients who presented with painless hematuria; microscopic (n=108) and macroscopic (n=65) were asked to participate. Only those patients who had an established diagnosis of hematuria (>10 red blood cells in the urinalysis) were included in the study. Patients with microscopic hematuria were all referred by internists. Their complaints were a sensation of burning during urination, lower urinary tract symptoms, loss of weight, anemia and persistent fever. Patients with painless macroscopic hematuria had no other symptoms. Patients eligible for the study were those with a new diagnosis of hematuria who were able to undergo cystoscopy. Patients who did not agree to undergo cystoscopy as well as those with an established history of recurrent superficial bladder cancer and/ or carcinoma in situ were excluded from the study. Eligible patients had a urinary tract abdominal ultrasound examination performed with a full bladder. Urinary tract ultrasonography was performed by two consultant radiologists. The examination was performed with a convex 2.5-5 MHz transducer. A linear 5-7.5 MHz transducer was also used to visualize defects of the bladder dome. (LOGIQ 3 Pro, General Electric Ultraschall, Beethovenstraße 239, Solingen, Germany). Patients who presented with blood casts in the urine sample underwent transurethral catheterization and bladder lavage. Eight patients who presented with serious hematuria that did not respond to bladder lavage underwent Color or spectral Doppler ultrasound investigation. The bladder was examined with several different transducers to maximize the likelihood of detection of small lesions. High frequency curved arrays were used to scan the bladder dome. Duplex/CFD was used to determine if a bladder mass was vascularized and therefore arising from the wall in some cases. The main diagnostic criterion for the ultrasonographic diagnosis of superficial bladder cancers was the presence of irregular soft tissue structures of low- to intermediate-echo texture projecting into the bladder lumen from a fixed mural site. Two consultant urologists, who were blinded to the ultrasound results, with an Olympus rigid cystoscope performed cystoscopy. The two urologists performed cytoscopy on the same patient in the two different sesions, and therefore were not aware of the results of the other. A uniform registration form was used for recording findings of ultrasound and cystoscopy from each patient. At the end of the study, the reported findings of urinary tract ultrasonography were correlated with those visualized by cystoscopy. The tolerability of cystoscopy was self-estimated and recorded: each patient was provided with an optical pain-meter scaled from 0 to 10 to measure discomfort or pain level of cystoscopy. Pain-Meter is a self-administered pain assessment tool developed in 1996 for the purposes of improving assessment and management of pain in acute and chronic pain patients. The Pain-Meter (Lavipharm, Greece) used in our study is a hard, white tool that consist of a 10-cm visual analogue scale with a moveable marker that patients used to rate their pain.^9^ Patients with ultrasound and/or cystoscopy suspicious for bladder carcinoma were further evaluated. Transurethral resection of the tumors and histopathological analyses were performed at Urology Department, Thriassio General Hospital, Elefsina, Greece and pathology Urology Department, Thriassio General Hospital, Elefsina, Greece respectively. Confirmation of the bladder carcinoma was achieved by histopathogical examination of the submitted specimens of bladder biopsy in each case. The locally appointed ethics committee approved the research protocol, while all subjects were informed and consented to participation. The administration of the General Hospital of Thebes provided financial support.

## RESULTS

Of 148 patients (53 women and 95 men) finally included, all underwent ultrasound examination. Twenty-five patients were excluded from the study (13 refused cystoscopy, 3 were not suitable for undergoing cystoscopy, 7 had a history of recurrent bladder cancer and 2 were under treatment for carcinoma in situ). The male patients ranged in age from 33 to 85 years (median 63.2 years) while the female patients ranged in age from 34 to 76 years (median 58.6 years). The examination was interrupted in 7 of 40 patients due to limited tolerability of the cytoscopy procedure while the remaining 34 did not attend the second cytoscopy session. Thirty-nine patients were finally identified with bladder carcinoma by cystoscopy. In 34 patients (87.1%) ultrasonography accurately diagnosed tumor while in 5 patients it failed to clearly diagnose bladder carcinoma. More precisely, in one patient the bladder carcinoma was reported as a prostatic lobe and in another patient the tumor was located in a bladder diverticulum and was reported as a vesical stone. In the remaining three cases the bladder tumor was smaller than 3 mm. Two patients had a false-positive ultrasonographical diagnosis of bladder cancer. In both patients the intraluminal-filling defect was the median lobe of the hypertrophic prostate. In two cases, radiologists suggested different diagnoses (interobserver variation). Inter-observer variations recorded in the ultrasonography registration form were mainly differences in estimating the size and number, in the case of multiple tumors, as well as the localization of bladder tumors (Table 1).

**Table 1:** Misdiagnoses of ultrasonography and cystoscopy in the detection of bladder carcinoma.

	Abdominal Ultrasound	Cystoscopy
Interobserver variations	2	2
False positive findings	2	1
False negative findings	5	1

The performance characteristics of ultrasonography in the diagnosis of bladder carcinoma are shown in Table 2. In 6 of the 39 patients with bladder carcinoma, an abnormality in the upper urinary tract was found (hydronephrosis and hydroureter secondary to ureteric involvement by bladder carcinoma), as well as renal pelvis stones and renal cysts. In two cases, interobserver variations in the cystoscopy registration form were only in the exact tumor size. In the case of carcinoma in situ one of the two urologists did not identify the presence of the tumor. Abnormalities resembling bladder cancer were described. Final diagnosis of cancer was established upon pathologic examination of the resected tissue.

**Table 2:** Evaluation of ultrasonography in the diagnosis of bladder carcinoma.

	Actual diagnosis		
Result	With disease	No disease	Totals
Positive	34	2	36
Negative	5	103	108

**Totals**	**39**	**105**	**144**

Sensitivity=87.1% (34/39), specificity=98.1%(103/105). Positive predictive value=94.4%(34/36), negative predictive value=95.4%(103/108).

The mean discomfort level of cystoscopy estimated by each patient on optical pain-meter was 5.74. This value was slightly higher when calculated only for men and slightly lower when calculated only for women. Differences in cystoscopy tolerability (ranged from 0-8 in males, 4-10 in females) between males and females were not of statistical significance.

## DISCUSSION

Cystoscopy is currently the best test for evaluation of the bladder as a possible source of hematuria; however, as seen in our study, in rare cases cystoscopy may not reveal a tumor in the bladder.^2^ Additionally, diagnostic cystoscopy is usually performed on an outpatient basis under local anaesthesia and is usually considered a painful diagnostic procedure. However, the tolerability and acceptance of diagnostic cystoscopy by patients has not been thoroughly documented. As seen from our study, the pain is moderately tolerated. Our findings are comparable to those of a few previous studies examining the tolerability and morbidity of other endoscopic procedures (eg. ureteroscopic lithotripsy under local anaesthesia, urodynamic studies etc).^4^–^7^ In previous studies, differences in cystoscopy tolerability among male and female patients were attributed in these studies to the normal anatomical difference between the male and female urethra as well as by the additional difficulties in performing cystoscopy in male patients with enlarged prostates. Moreover, since routine pelvic gynecologic exams are usually performed annually among women of childbearing years it is plausible that women feel less discomfort than men when undergoing cystoscopy.

Technical and scientific advances in radiology have increased the diagnostic accuracy of imaging methods in the investigation of hematuria and various authors have proposed the use of imaging methods as the sole initial investigation for detection of bladder carcinomas in patients presenting with hematuria.^3^ In fact, transabdominal ultrasonography is a simple and quick examination that can be safety performed on all individuals with no restrictions (for example, the elderly and handicapped patients that cannot undergo cystoscopy, and septic patients and those with renal failure that are contraindicated for intravenous pyelography). Ultrasonography is also easily available, cost-effective, and non-invasive, requiring no special preparation, and provides images of both the upper and lower renal tract. In the present study, ultrasonography accurately detected 87.1% of bladder carcinomas, while in 3 of 39 patients with bladder carcinoma, abnormalities not related to bladder carcinoma were also found, including renal pelvis stones (n=2), and renal cysts (n=1). In the past, the accuracy of ultrasound devices in the diagnosis of superficial bladder tumors was less than that of the current devices and this led to an underestimation of the value of ultrasound; thus, ultrasonography has been accused of not always being an appropriate method for the diagnosis of bladder cancer. Technological evolution has rendered current scanners, which combine several different transducers and color or spectral Doppler imaging facilities, more accurate in the visualization of intra-luminal filling defects of the bladder. In fact, detection rates for bladder carcinoma have been increased from 82% to 95%.^10^,^11^ Despite remarkable improvements in the diagnostic accuracy, some of the pitfalls of ultrasonographic tests for evaluation of the bladder as a possible source of hematuria still remain. Smaller lesions (smaller than 0.5 cm) and lesions located in the dome or bladder neck are more difficult to visualize sonographically. Tumor configuration is also an important factor: plaqueilike lesions are almost certainly harder to detect than polypoid ones.^11^ Notably, in our study, the smallest carcinoma detected was 4 mm in size, while in 3 of 5 cases the ultrasound failed to detect bladder carcinoma for tumor sizes smaller than 3 mm. Note that the ultrasonography might lead to misdiagnosis due to external factors, such as obesity of patient and degree of bladder distension, while its adequacy depends on the experience and skill of the person performing the study.^10^

According to our findings, despite remarkable improvements in diagnostic accuracy, ultrasonography is still inferior to cystoscopy in the evaluation of the bladder as a possible source of hematuria and neither ultrasound nor the combination of urinary cytology and ultrasonography can replace the standard cystoscopic examination.^12^

Since the diagnosis of bladder cancer requires histopathological confirmation (on the core biopsy obtained during cystoscopy or of the bladder specimens obtained after Transurethral resection of the tumor) ultrasonography is certainly not the most adequate examination.

However despite its own pitfalls it can provide a map of suspected areas to be further assessed by the following cystoscopy, while, the visualisation of a bladder tumour in earlier imaging -especially in small health settings where cystoscopy is not available - can save money and time. On the perspective of the authors, while endoscopic approach and biopsy remains the gold standard, ultrasonography represents a valuable tool in the initial radiological investigation for detection of bladder carcinomas. Patients presenting with painless hematuria should be initially investigated by ultrasound and -only if necessary-by cystoscopy in order to reach a complete diagnosis.
